# Preparation, Characterization and Pharmacodynamic Evaluation of Fused Dispersions of Simvastatin using PEO-PPO Block Copolymer

**Published:** 2012

**Authors:** Harjeet Singh, Betty Philip, Kamla Pathak

**Affiliations:** a *Department of Pharmaceutics, School of Pharmacy, College of Pharmacy and Nursing, University of Nizwa, Birkat Al Mouz, Nizwa 616, Sultanate of Oman.*; b *Department of Pharmaceutics, Rajiv Academy for Pharmacy, Mathura, Uttar Pradesh, India.*

**Keywords:** Lutrol NF 127 prill surfactant, Inverse thermo sensitivity, %Crystallanity Index, Optimization, 32 Factorial design.

## Abstract

The solubility enhancement of poorly soluble compounds is an important task in pharmaceutical technology as it leads to better bioavailability and a more efficient application. Fused dispersions (FDs) of simvastatin (SIM) using PEO-PPO block copolymer were prepared which paved the way for the formation of an amorphous product with enhanced dissolution and bioavailability. The accumulative solubility of simvastatin (SIM) from PEO-PPO block copolymer (Lutrol NF 127 prill surfactant) was found to be superior to the drug alone which may be due to the increased oxyethylene content that played the major role in solubility enhancement. A 3^2^ full factorial approach was used for optimization wherein the temperature to which the melt-drug mixture cooled (X_1_) and the drug-to-polymer ratio (X_2_) were selected as the independent variables and the time required for 90% drug dissolution (t_90%_) was selected as the dependent variable. A low level of X_1_ and a high level of X_2_ were suitable for obtaining higher dissolution of SIM from SIM FDs. On increasing melt to cool drug temperature, t_90%_ increased thus improving dissolution rate of FD_2_ batch with the maximum drug release (99.63%) in 120 min. The optimized FDs were characterized by saturation solubility study, drug content, *in-vitro* dissolution, fourier transform infrared spectroscopy, scanning electron microscopy, differential scanning calorimetry, x-ray diffraction, ^1^HNMR spectroscopy and pharmacodynamic evaluation. Capsules containing optimized FDs were prepared and compared with marketed brand (SIMVOTIN®). Finally, it can be concluded that the optimized FDs of SIM ameliorate the solubility and dissolution of drug with improved pharmacodynamic activity.

## Introduction

The solubility of poorly soluble drugs for the purpose of improving their pharmaceutical and biological availability still remains one of the major technological problems. Drug release is a crucial and rate limiting step for oral bioavailability, particularly for drugs with low gastrointestinal solubility and high permeability (1). By improving the drug release profile of these drugs, it is possible to enhance their bioavailability and reduce their side effects (2-4). Nearly 40% of all new drug candidates are classified as poorly soluble, which makes it difficult to develop the pharmaceuticals formulations (5). Solid dispersions are one of the most successful strategies to improve the drug release of poorly soluble drugs (6, 7). These can be defined as the molecular mixtures of poorly water-soluble drugs in hydrophilic carriers, which present a drug release profile that is driven by the polymer properties (8).

Simvastatin (SIM) is cholesterol lowering agent and widely used to treat the hypercholesterolemia. It is practically insoluble in water. Such drugs often show dissolution after the oral administration as the rate-limiting step for their *in-vivo* absorption and the appearance of pharmacological effect. Therefore, improvements in solubility and/or dissolution rate of poorly water-soluble drugs may lead to the enhancement of their bioavailability (9, 10).

PEO-PPO block copolymers (Lutrol NF 127 prill surfactant) are often considered as “functional excipients” since they are essential components and play an important role in a formulation. They are extensively used to increase the solubility and to improve the bioavailability of poorly water-soluble drugs (11). The polyoxyethylene segment of Lutrol NF 127 prill surfactant is relatively hydrophilic, while the polyoxypropylene segment is relatively hydrophobic. The Lutrol NF 127 prill surfactant is applied in a growing trend in the formulation of dosage forms owing to their low toxicity and ability to form a clear solution or gel in aqueous media and have consequently solubilized many water-insoluble compounds essentially by the formation of micelles (12).

In the present study, fused dispersions of simvastatin (SIM) with hydrophilic carrier Lutrol NF 127 prill surfactant were prepared and a 32 full factorial design approach was used for the optimization of process variables. The aim of the present work was to study the joint influence of the independent variables (viz. temperature) to which the melt-drug mixture cooled (X_1_), and the drug to- polymer ratio (X_2_) on the dependent variable of t_90%_ (time required for 90% drug dissolution) in fused dispersions. The physical mixture (PM) was prepared in equimolar ratio and used for characterization. Physicochemical characterization was performed to evaluate the occurrence of chemical interaction between the drug and carrier. In addition, the improvement in rate and extent of *in-vitro* drug release from FD’s was justified by the pharmacodynamic evaluation study in rats.

## Experimental

Simvastatin USP was generously donated by Krebs Biochemical’s and Industries Pvt Ltd.

(Hyderabad, India), Lutrol NF 127 prill surfactant (BASF,U.S.A), Polyvinyl pyrrolidone K29/32 (Acros organics, U.S.A) and Glyceryl monostearate LR were supplied by CDH Ltd (New Delhi, India). All other chemicals and solvents were of analytical grade. Double distilled water was used throughout the studies.

*Preparation of fused dispersions*

The dispersions of drug and carrier were prepared by the fusion method. Carrier was heated at a temperature of 55°C ± 0.5°C using a thermostatically controlled water bath. The drug in a 1:2, 1:4 and 1:6 the drug to polymer ratio was dispersed in the molten carrier. The resulting mixture was immediately cooled to 10°C, 20°C and/or 30°C using an ice-water mixture (according to the factorial design Table 1) and maintained at the specified temperature for 2 h. The solidified mass was then removed from the ice-water mixture and allowed to attain the room temperature. It was stored at the room temperature for 24 h and then pulverized using a glass mortar pestle. The pulverized mass was sifted through a # 40 sieve, weighed, and transferred to amber the colored glass vials for storage and the yield was determined using the following formula:

Yield = (a / b + c) × 100

Here, a is the weight of the fused dispersion sifted through a # 40 sieve, b is the weight of drug taken for fused dispersion preparation, and c is the weight of carrier taken for fused dispersion (13).

*Experimental design*

A 3^2^ full factorial design (Tables 2 and 3) was employed to systematically study the joint influence of the independent variables’ effect, temperature at which the melt-drug mixture cooled (X_1_ ), and the drug-to-carrier ratio (X_2_) on the dependent variable t_90%_ (time required for 90% drug dissolution). In this design, two factors were evaluated, each at three levels, and experimental trials were performed for all nine possible combinations. A statistical model incorporating interactive and polynomial terms was used to evaluate the response (14).

Y = b_0_ + b_1_X_1_ + b_2_X_2_ + b_12_X_1_X_2_ + b_11_X_12_ + b_22_X_22_

Here, Y is the dependent variable, b_0_ is the arithmetic mean response of the nine runs and b_1_ is the estimated coefficient for the X_1_ factor. The main effects (X_1_ and X_2_) represent the average result of changing one factor at a time from its low to high value. The interaction terms (X_1_X_2_) show how the response changes when 2 factors are simultaneously changed. The polynomial terms (X_12_ and X_22_) are included to investigate the nonlinearity. The composition of the factorial design batches (FD_1_ to FD_9_) are shown in Table 1.

**Table 1:** Composition of 32 factorial design.

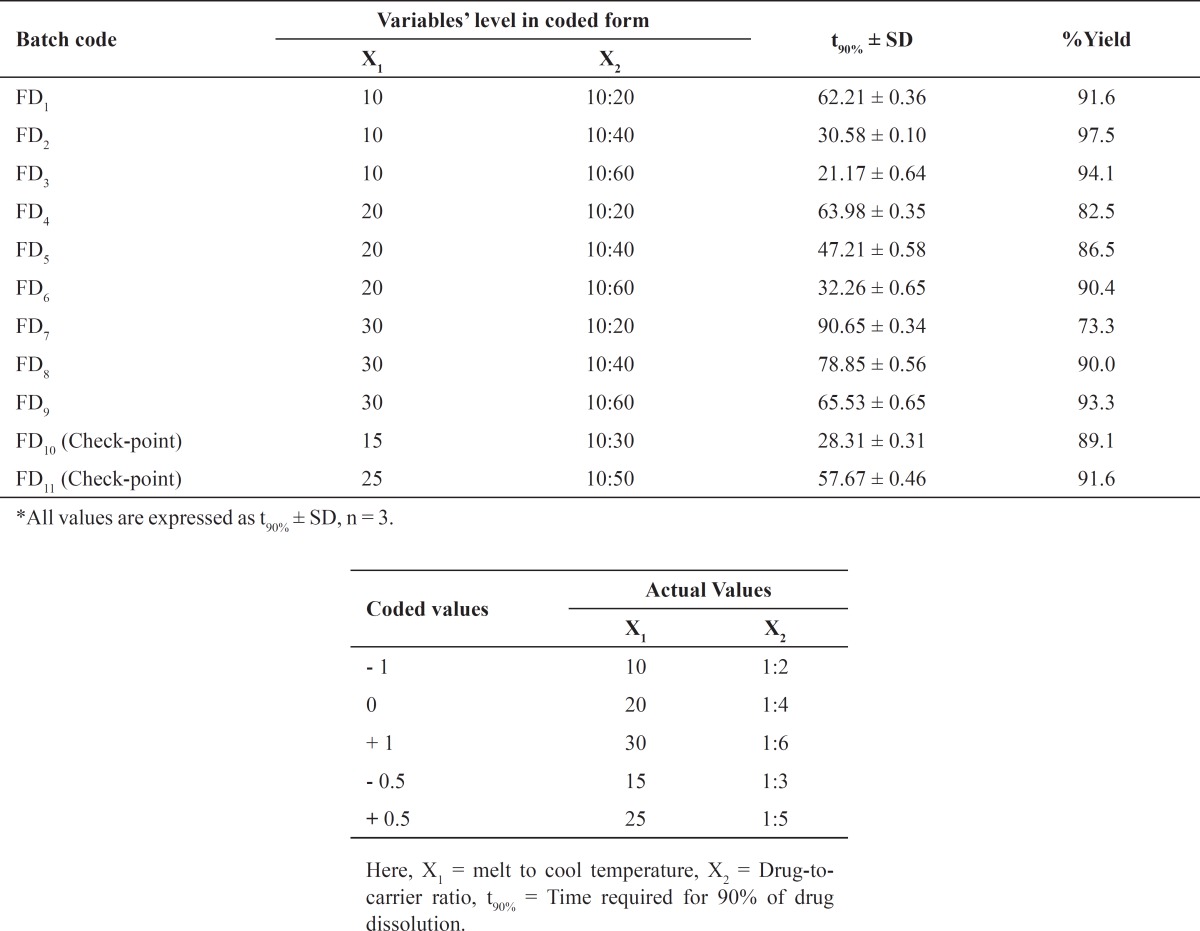

*In-vitro dissolution studies*

*In-vitro* dissolution of fused dispersions and PM were performed using USP XXIV Apparatus I in 900 mL of phosphate buffer (pH = 6.8) at an agitation rate of 100 rpm. The temperature of medium was maintained at 37 ± 0.5°C. Ten mg of drug or its equivalent weight of the prepared solid system was taken and analyzed for the dissolution. A 5.0 mL sample was withdrawn at specific time-points over a 2 h period and equal volume of fresh dissolution medium was used to maintain a constant volume. The aliquot samples were filtered and the drug concentration was determined through the ultraviolet method at 239 nm. The dissolution experiments were conducted in triplicate.

*FT-IR studies*

The FT-IR spectra of the pure drug, physical mixture, carrier and formulation FD_2_ were recorded with the shimadzu FTIR-8400 spectrometer (Shimadzu, Japan). The samples were prepared through the potassium bromide disc method and scanned for the absorbance of 4000-400 cm^-1^.

**Table 2:** Results of regression analysis

**Response t**_90%_	**b**_0_	**b**_1_	**b**_2_	**b**_11_	**b**_22_	**b**_12_
**Full model (FM)**	44.71	18.45	- 18.87	12.77	17.21	1.76
**Reduced model (RM)**	44.71	18.45	- 18.87	12.77	17.21	-

indicates b_12 _term is omitted in reduced model

*Differential scanning calorimetry (DSC)*

Thermograms of pure drug, physical mixture, carrier and formulation (FD_2_) were recorded on a Perkin–Elmer (Pyris Diamond) model differential scanning calorimeter. About 10 mg of samples were sealed in aluminum pans and heated at a rate of 10°C/min from 30 to 300°C under the nitrogen atmosphere flow rate of 20 mL/min.

*X-Ray powder diffractometry (XRD)*

Powder X-ray diffraction patterns of pure drug, physical mixture, carrier and formulations (FD_2_, FD_5_ and FD_8_) were recorded with a D-8 Advance SRD-BRUKER (Germany) using a copper target, voltage of 40 kV and current of 30 mA, at a scanning speed of 5°C/min. Percentage Crystallanity index (C.I.) was calculated through the following Equation:

%Crystallanity index = I_020_ - I_am_/ I_020_ × 100 Equation (1)

Where, I_020_ is intensity at 20°; I_am_ is the lowest when 2θ is near the 8°.

*Scanning electron microscopy (SEM)*

The surface morphology of pure drug, physical mixture, carrier and formulations (FD_2_, FD_5_ and FD_8_) were examined using Scanning Electron Microscope (LEO 435 VP, UK). The samples were fixed on a brass stub using double-sided tape and then gold coated in vacuum through a sputter coater. The pictures were then taken at an excitation voltage of 15 k.

^1^ *HNMR spectroscopy*

To determine the nature of proton or protonated group in the pure drug, carrier and FD_2_ formulation, the ^1^HNMR spectrum in DMSO were recorded on Bruker Advance II (DRX-400, Japan), FT-NMR spectrometer, 300 MHz, using TMS as internal standard, chemical shift (*δ*) were recorded in ppm.

*Pharmacodynamic evaluation*

The hypolipidemic activity of FD was determined in comparison with pure simvastatin in healthy albino rats of either sex or weighing between 150-200 g. The animals were procured from animal house, Rajiv Academy for Pharmacy, Mathura, India. General and environmental conditions were strictly monitored. Animal handling routines were performed according to the Good Laboratory Practice. Animals had free access to food and water ad libitum. The animal experimentation was approved by the Institutional Animal Ethics Committee of Rajiv Academy for Pharmacy, Mathura, India (Registration No.882/ac/05/CPCSEA).

The animals were divided into three groups of Control, Reference and Test, each of which consisted of 4 animals and the treatment was given for 14 days. Each group daily received 1 mL of coconut oil orally by oral feeding needle. Reference and test groups additionally received aqueous suspensions of pure drug and FD_2_ respectively (equivalent to 10 mg/Kg body weight), prepared using 2% w/v carboxymethyl cellulose (CMC) as a suspending agent. Blood samples were collected through retro orbital puncture or from tail vein initially, after 7 and 14 days. Samples were analyzed for total cholesterol, triglycerides (TG), low density lipoproteins (LDL), very low density lipoproteins (VLDL) and high density lipoproteins (HDL) cholesterol levels by *in-vitro* diagnostic kit (RECKON BIOMEDICAL PVT.LTD). Statistical analysis was done for the determination of differences in lipid profiles of treatment and control groups by unpaired t-test and p < 0.05 was taken as significant.

**Table 3:** Serum lipid profiles of various experimental groups at different time intervals

**Group**	**Time (in days)**	HDL (mg/dL)*	LDL (mg/dL)*	TG (mg/dL)*	TC (mg/dL)*	VLDL (mg/dL)*
**Control group**	0	22.3 ± 2.66	26.7 ± 1.32	72.8 ± 3.67	51.4 ± 2.95	15.3 ± 1.17
	7	41.4 ± 3.02	36.3 ± 1.96	146.3 ± 2.96	72.8 ± 2.36	20.5 ± 1.23
	14	46.8 ± 3.01	49.3 ± 1.82	189.3 ± 3.05	89.1 ± 2.42	23.5 ± 1.33
**Reference group**	0	22.9 ± 3.12	39.2 ± 1.29	72.4 ± 5.90	47.1 ± 1.25	13.6 ± 1.12
	7	44.6 ± 2.85	34.1 ± 1.19	112.4 ± 4.32	43.5 ± 1.16	12.4 ± 1.09
	14	49.2 ± 2.74	28.4 ± 1.20	186.7 ± 3.87	41.2 ± 1.48	11.3 ± 1.21
**Test group**	0	22.2 ± 2.10	38.6 ± 1.24	71.6 ± 3.45	50.1 ± 4.74	14.3 ± 1.09
	7	47.3 ± 1.89	33.1 ± 1.35	110.7 ± 2.52	41.4 ± 4.38	11.2 ± 1.21
	14	53.2 ± 1.76	20.1 ± 1.48	134.6 ± 2.43	40.7 ± 4.16	9.9 ± 1.24

*Mean ± SD, n = 3, p < 0.05

## Results and Discussion

*In-vitro dissolution studies*

**Figure 1:**
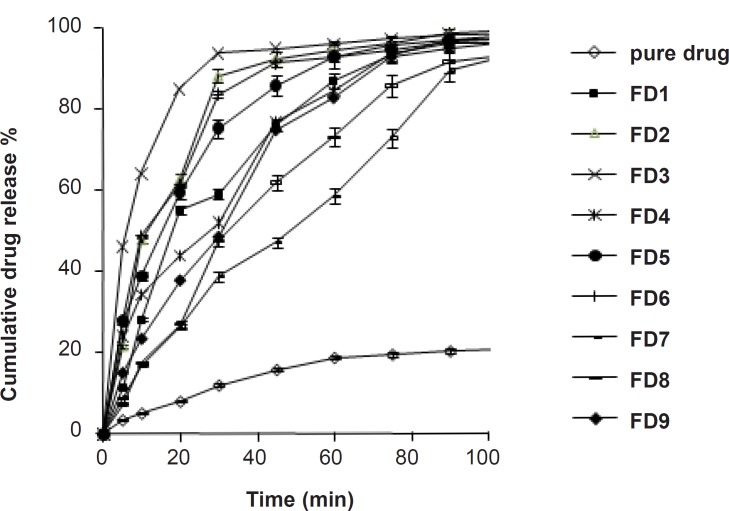
Comparative *in-vitro* dissolution profile of pure drug and different formulations in phosphate buffer (pH = 6.8)

**Figure 2:**
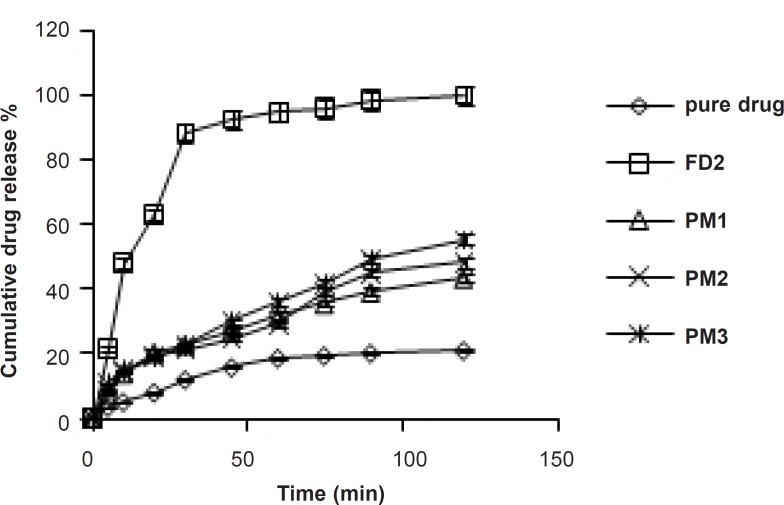
Comparative *in-vitro* dissolution profile of pure drug, FD_2_ and physical mixtures in phosphate buffer (pH = 6.8).

The dissolution profile of pure simvastatin was extremely low, with only 21.04% of drug release during 120 min of dissolution run in phosphate buffer (pH = 6.8), which might be attributed to the floating of the drug on the surface of dissolution medium (15). Fused dispersions of simvastatin showed enhancement of drug dissolution due to the conversion of simvastatin into a less crystalline and/or amorphous form. The improved dissolution rate was observed in all the prepared system and maximum release was seen in FD_2_ batch (99.63% in 120 min) prepared by the fusion method as shown in Figure 1. PMs presented slight improvement in drug release which could be attributed to the improved wettability of drug particles by the presence of hydrophilic amorphous carrier as shown in Figure 2. The model dependent parameter t_90%_ obtained from the cumulative percentage drug released is shown in Table 1. The t_90%_ for all the nine batches (FD_1_ to FD_9_) displayed a wide variation from 90.65 to 21.17 min. The data clearly indicates that X_1_ and X_2_ strongly influence the t_90%_. From the data, it was observed that on increasing melt to cool drug temperature, t_90%_ was increased. As the temperature was decreased, the amount of dissolved drug was increased, which may be attributed to a higher energy state of drug particles at low temperature, resulting in a more amorphous form. The superior solubility of drug at low temperatures can further be attributed to the inverse thermosensitivity of Lutrol NF 127 prill surfactant which is soluble at low temperature (but gels are soluble at higher temperature). The increased dissolution rate and bioavailability for fused dispersions (FDs) of simvastatin (SIM) using PEO-PPO block copolymer may also be attributed to the increased oxyethylene content which played a major role in the solubility enhancement. This concept is further discussed in XRD studies.

*Validation of experimental design*

Preliminary investigations of the process parameters revealed that X_1_ and X_2_ factors highly influenced the rate of *in-vitro* dissolution and hence, were used for the further systematic studies. As stated in the previous section, X_1_ and X_2_ strongly influence the t_90%_. A polynomial equation was constructed that it would relate the effect of individual factor and the interactions between the factors through coefficients in the polynomial equation generally calculated for a response (in this case, t_90%_).

Y = 44.71 + 18.45 X_1_ – 18.87X_2_ + 1.76X_1_X_2_ + 12.77 X_12_ + 17.31X_22_

The transformed Equation was:

Y = 44.71 + 18.45X_1_ – 18.87X_2_ + 12.77 X_12_ + 17.31X_22_

The fitted polynomial equations (full and reduced model) relating the response t_90%_ to the transformed factors are shown in Table 2. The polynomial equations can be used to draw the conclusions after considering the magnitude of coefficient and the mathematical sign it carries (positive or negative). The significance level of coefficient b_12_ was found to be p = 0.3765 and hence it was omitted from the full model equation to generate the reduced model equation. Table 2 shows the results of regression analysis. The coefficients b_1_, b_2_, b_11_, and b_22_ were found to be significant at p < 0.05 and thus, were retained in the reduced model. Multiple linear regression analysis (Table 2) revealed that coefficient b_1_ is positive and b_2_ is negative. This indicates that on increasing X_1_, t_90%_ is increased. It was observed that by decreasing the temperature, the amount of dissolved drug is increased, which may be attributed to a higher energy state of drug particles at low temperature, resulting in a more amorphous form. The released studies of batches with increasing concentration of Lutrol NF 127 prill surfactant (X_2_) revealed that as the concentration of Lutrol NF 127 prill surfactant is increased, t_90%_ is decreased (Table 2). This could be due to the fact that simvastatin may exist in the fused dispersion in two different forms, namely crystalline and amorphous. The rate of drug dissolution from the fused dispersion depends on the proportion of two forms, which inturn depends on the proportion of Lutrol NF 127 prill in the fused dispersion. As the weight fraction of Lutrol NF 127 prill is increased, the proportion of the amorphous form of simvastatin may be increased, which inturn results in the enhancement of simvastatin dissolution (16). Check-point batches FD_10_ and FD_11_ were prepared at X_1_ = - 0.5 and + 0.5 and X_2_ = + 0.5 and - 0.5 levels, respectively (Table 1). Yates algorithm was used for the calculation of predicted response (Y_90%_). The theoretical t_90%_ of batches FD_10_ and FD_11_ were 26.39 and 58.46 min, respectively. The experimental values are 28.31 and 57.67 min (Table 1), which are in good agreement with the theoretical values. Formulations FD_2_ and FD_3_ that exhibited the least t_90%_ values, were analyzed for the selection of optimized formulation. The t_90% _of both these batches were almost similar (Table 1) and exhibited an insignificant difference as confirmed by Student t-test (tcal = 0.979, ttab = 2.78). However, the %yield of FD_2_ was higher as compared to FD_3_. This guided the selection of FD_2_ as the optimized batch for the dissolution enhancement of simvastatin which was further subjected to the spectral characterization and pharmacodynamic activity.

*FT-IR studies*

**Figure 3:**
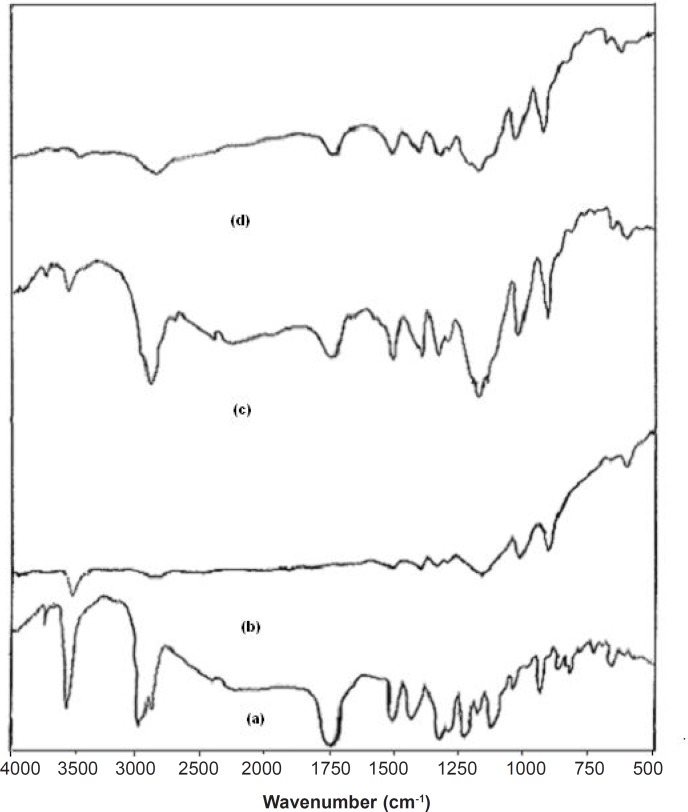
FT-IR spectra of (a) Simvastatin, (b) Lutrol NF 127 prill surfactant, (c) Physical mixture, and (d) FD_2_

FT-IR studies were done to detect the possible interactions between the SIM and carrier (Lutrol NF 127 prill surfactant) in the fused dispersions leading to the amorphous state of simvastatin shown in Figure 3. The FT-IR spectra of pure simvastatin presented characteristic peak at 3552 cm^-1^ and 3749 cm^-1 ^(O−H stretch vibration), 2960 cm^-1^ (C−H stretch vibration) and 1730 cm^-1^, and 1164 cm^-1^ and 1066 cm^-1^ (stretch vibration of −C−O and −C=O carbonyl functional group). The spectrum of Lutrol NF 127 prill surfactant showed important bands at 3634 cm^-1^ (O−H stretch vibration) that was attributed to the presence of water confirming the broad endotherm detected in DSC experiments and 1282 cm^-1^ (−C−O stretch vibration). The FT-IR spectra of physical mixture (PM) seemed to be only a summation of drug and carrier. This result suggested that there were no interactions between the drug and carrier in PM and simvastatin maintained its crystallanity as observed in thermal analysis. If the drug and carrier interact, then the functional groups in the FT-IR spectra will show band shifts and broadening compared to the spectra of drug and carrier. Initial characterization of FDs by FT-IR studies indicated band shifts and broadening compared to the spectra of drug and carrier. In the FD_2_ formulation, band shifts observed at 1714 cm^-1^, 3446 cm^-1^ and 3629 cm^-1^ and broadening at 1165 cm^-1^, suggested intermolecular hydrogen bonding via the −C=O group of simvastatin and O−H group of Lutrol NF 127 prill surfactant. Thus, a combination of interaction and decreased mobility of simvastatin during the preparation of fused dispersions may be the cause of stable amorphous form of drug inside the carrier (17).

*DSC studies*

**Figure 4:**
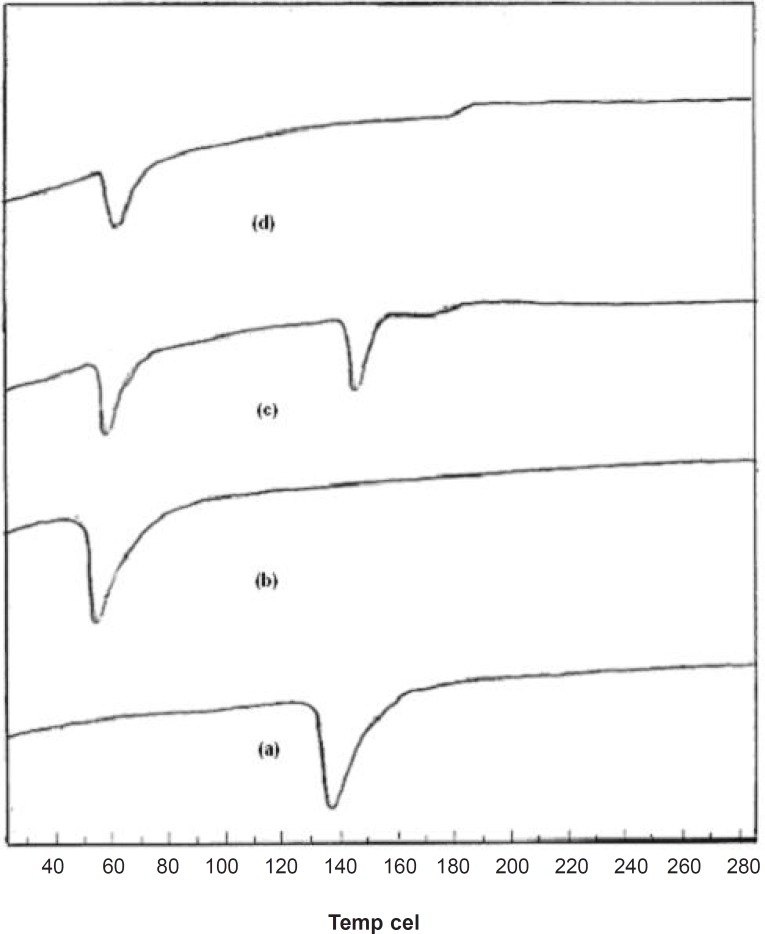
DSC thermograms of (a) Simvastatin, (b) Lutrol NF 127 prill surfactant and (c) Physical mixture, (d) FD2

Supporting evidence for the fused dispersions was also obtained from DSC studies. DSC thermograms elicited significant suppression of carrier endothermic peak in FD_2_, suggesting a homogeneous dissolution of the drug in carrier. From the thermogram, it was seen that a sharp endothermic peak corresponding to the melting point of crystalline pure drug was found at 139°C and for pure carrier at 58°C. The thermogram of the PM was merely a combination of thermogram of pure drug and carrier as shown in Figure 4. The endothermic peak corresponding to melting of pure dug was absent in the DSC thermogram of fused dispersion of FD_2_. It might be due to the presence of the amorphous form of pure drug in the fused dispersion or the dissolution of crystalline simvastatin into the molten carrier (18). DSC thermograms of pure compounds, PM and FD_2_ are shown in Figure 4.

*XRD studies*

**Figure 5:**
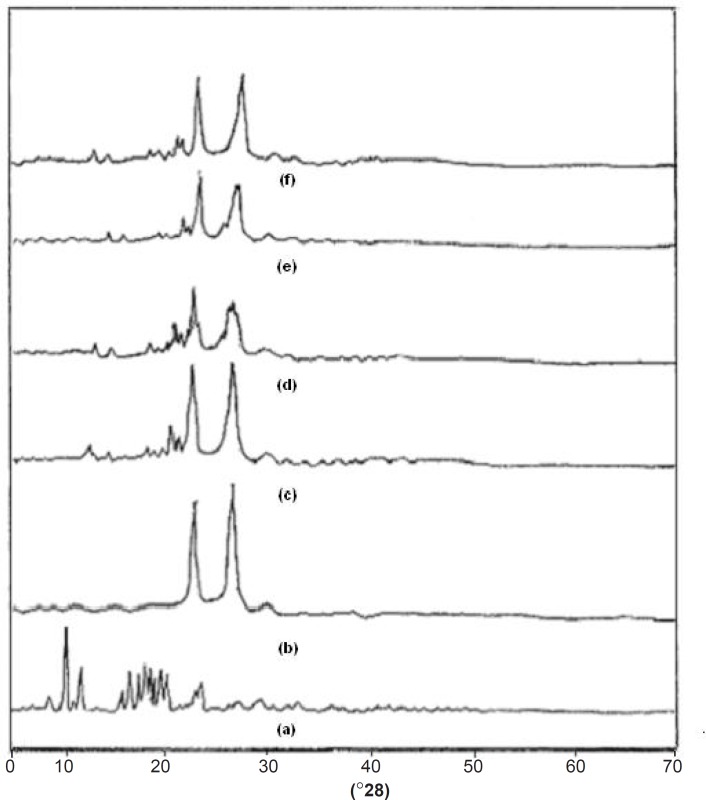
XRD spectra of (a) Simvastatin, (b) Lutrol NF 127 prill surfactant, (c) Physical mixture, (d) FD_2_ (e) FD_5_ and (f) FD_8_

One of the most useful parameters for the characterization of a crystalline polymer is its degree of crystallanity (CI). Various methods have been developed for the determination of the crystallanity of polymers, one of which is x-ray diffraction analysis. The XRD pattern of pure drug showed numerous distinctive peaks in the region of 8 to 250 (2θ) (9.32, 10.88, 15.57, 16.51, 17.17, 18.75, 19.32, 22.49) that indicated the crystalline nature of simvastatin. Fused dispersions displayed all the peaks shown via the drug however, intensity of the peaks was markedly reduced with decreased d-spacing. It was observed that as the melt to cool temperature is increased, the shielding of peaks is decreased (FD_2_ > FD_5_ > FD_8_) whereas the intensity of peaks is increased (FD_8_ < FD_5_ < FD_2_). In case of pure carrier, the predominant peaks were observed at 19.08^0^ and 23.21^0^. The distinctive diffraction peaks of simvastatin in the physical mixture persisted as shown in Figure 5. FD_2_ exhibited more considerable diminution in diffraction peaks than the physical mixture. CI was found to be 71.57%, 59% and 66% for pure drug, pure carrier and physical mixture. The results also indicated that as melt to cool temperature is decreased the percentage crystallanity index decreased the FD2 (55.03%), FD5 (60.76%), and FD8 (63.71%). The significant decrease in the intensity of major SIM crystalline peaks may be due to the partial loss of crystallanity as compared with diffractograms of their corresponding PM, pure drug and FD5 and FD8 formulations. This suggested that the drug in fused dispersions is amorphous as compared to the pure drug. Hence, increased dissolution of drug was observed since an amorphous form dissolves at a faster rate owing to its higher internal energy and thermodynamic properties relative to crystalline materials (19). X-ray diffractograms of pure compounds, PM and FD_2_, FD_5_ and FD_8_ are shown in Figure 5.

**Figure 6:**
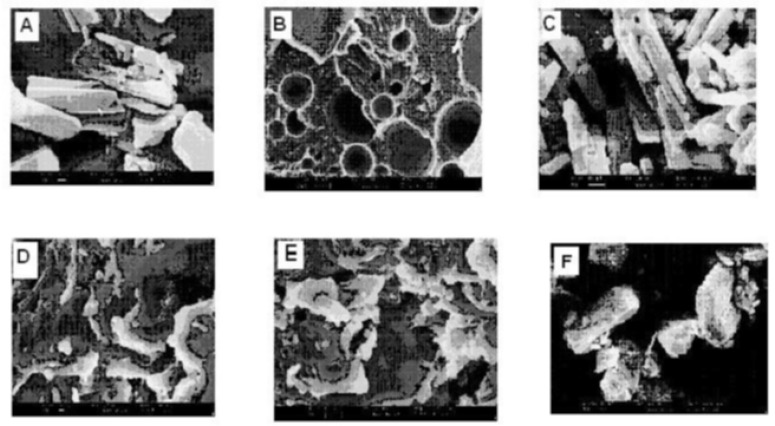
(A) SEM photomicrographs of Simvastatin, (B) Lutrol NF 127 prill surfactant, (C) Physical mixture, (D) FD_2_, (E) FD_5_ and (F) FD_8_

*SEM studies*

From scanning the electron photomicrographs, it was evidenced that the pure drug consisted of a mixture of some large crystals (8 to 10 mm) with microparticles, which might have been generated due to the micronization or any other size reduction process at the time of manufacturing. PM is seen as the combined characteristics of drug and Lutrol NF127 prill surfactant unlike the FDs where drug crystals were not possible to distinguish from carrier. FDs was formed at different melt to cool drug temperature revealed significant changes in particle shape and surface topography due to the impact of fusion process. SEM studies suggested that the homogeneous dispersions of drug in carrier may be due to the presence of amorphous state in FD_2_ formulation. FD_5_ and FD_8_ appeared as irregular shaped agglomerates with presence of few microcrystals, suggesting the possibility of residual crystallanity. FD_5_ and FD_8_ appeared as the irregular shaped agglomerates with presence of few microcrystals, suggesting the possibility of residual crystallanity. Slight surface smoothness was observed in FD_5_, as compared with FD_8_, which could be attributed to melt to cool drug temperature difference between them. FD_2_, on the other hand, looked like a smooth surface with very small particle size, suggesting the presence of amorphous state corroborating XRD observation (20). An XRD and SEM study confirms that as the melt to cool drug temperature is decreased, the amorphization of drug is increased. SEM images of pure compounds, PM and FD_2_ and also FD_5_ and FD_8_ are shown in Figure 6.

**Figure 7:**
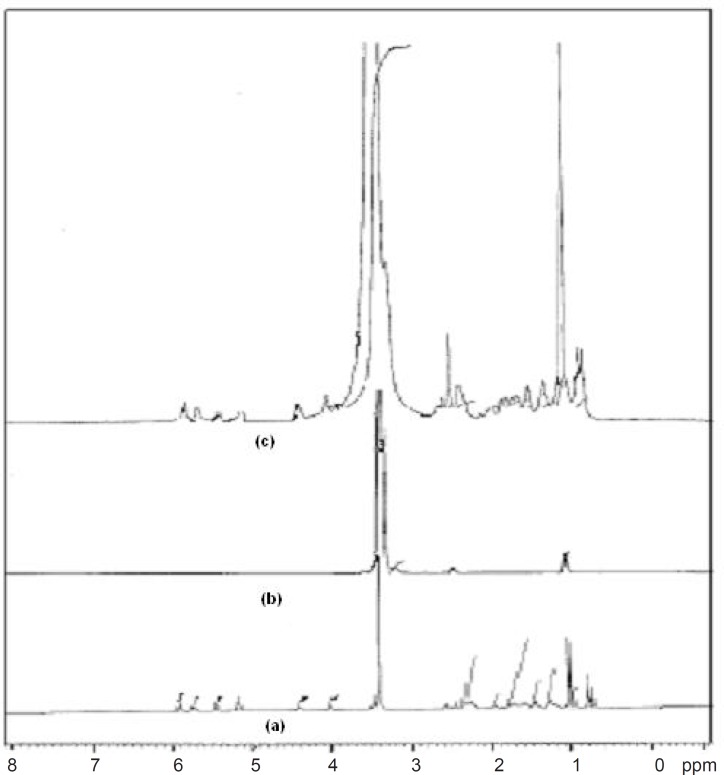
^1^HNMR spectra of (a) Simvastatin, (b) Lutrol NF 127 prill surfactant and (c) FD_2_

^1^ *HNMR spectroscopy*

^1^HNMR spectroscopy confirmed the results obtained from FT-IR studies. The ^1^HNMR spectrum of pure drug showed chemical shift from 5.930 to 5.962 ppm (d, ^1^H, C5H), 5.736 to 5.787 ppm (t, ^1^H, C4H), 5.481 ppm (s, ^1^H, C6H), 2.257 to 2.385 ppm (m, ^1^H, OH), 3.456 ppm (s, 10H, C3H, C7, C2, C3, C3”, C8, C9, C10, C2”, (CH3)2, 1.495 to 1.813 ppm (m, 6H, C3”, C7”), 0.991 to 1.037 ppm (m, 6H, C4”, C5’, C4’), 1.423 to 1.470 ppm (m, 2H, C5’), 3.304 to 3.710 ppm (m, 9H, CH3) and 2.494 ppm (s, ^1^H, OH). Pure carrier showed chemical shift from 3.493 to 3.710 ppm (m, 89H, CH2), 1.008 ppm (s, 4H, CH3) and 3.304 to 3.439 ppm (s, 2H, CH). FT-NMR (^1^HNMR) spectrum of formulation FD2 showed similar peaks of drug and carrier, 5.937 ppm (d, ^1^H, C5H), 5.589 to 5.767 ppm (t, ^1^H, C4H), 5.447 ppm (s, ^1^H, C6H), 2.301 to 2.388 ppm (m, ^1^H, OH), 3.462 to 3.729 ppm (m, 89H, CH_2_), 3.462 ppm (s, 10H, C3H, C7, C2, C3, C3”, C8, C9, C10, C2”, (CH_3_)_2_, 1.523 to 1.845 ppm (m, 6H, C3”, C7”), 0.980 to 1.022 ppm (m, 6H, C4”, C5’, C4’), 1.022 ppm (s, 4H, CH_3_) 1.445 to 1.489 ppm (m, 2H, C5’), 3.301 to 3.594 ppm (m, 9H, CH_3_) and 3.336 ppm (s, 2H, CH) which confirms the intermolecular hydrogen bonding between the drug and carrier (21). ^1^HNMR spectra are given in Figure 7.

**Figure 8:**
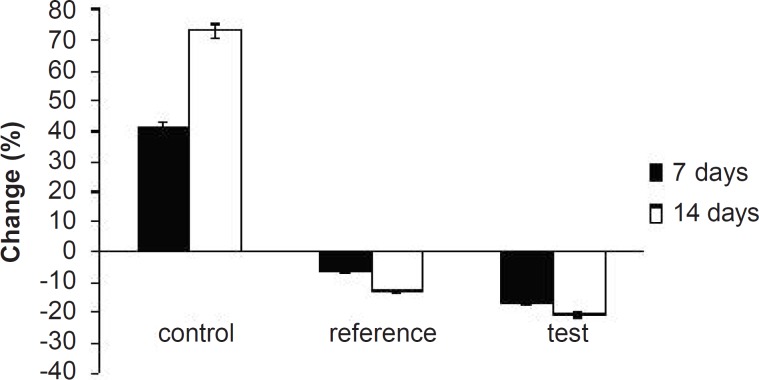
Percentage of changes in serum total cholesterol levels of experimental groups at different time intervals

**Figure 9:**
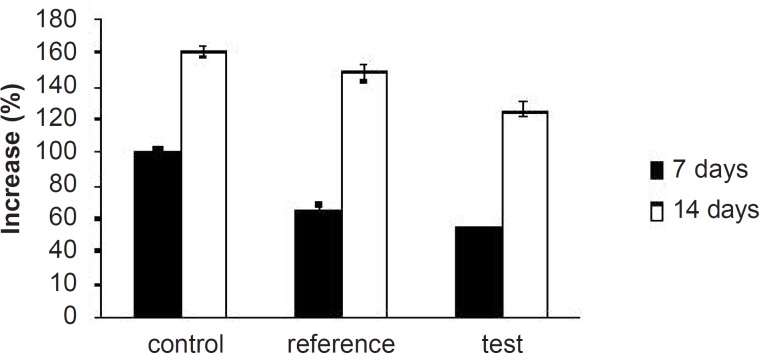
Percentage of increase in serum TG levels of experimental groups at different time intervals

*Pharmacodynamic evaluation*

Hyperlipidemia is an elevation of one or more of the plasma lipids, including cholesterol, cholesterol esters, triglycerides and phospholipids, in which statins play an important role for the treatment. Hyperlipidemic drugs like SIM (HMG-CoA reductase inhibitor) are known to reduce the elevated total cholesterol (TC), triglycerides (TG), LDL-cholesterol levels and VLDL-cholesterol levels in hyperlipidemic conditions. At the same time, they cause the elevation of HDL-cholesterol levels, which promote the removal of cholesterol from peripheral cells and facilitate its delivery back to the liver (22). This pharmacodynamic effect is reported to be dose-dependent hence, used as a basis for the comparison of *in-vivo* performance of pure SIM and FD_2_. The administration of excess coconut oil, which is a rich source of saturated fatty acids, promotes the biosynthesis of cholesterol in liver and leads to hypercholesterolemia. The serum lipid profiles of all the experimental groups at different time intervals are presented in Table 3. As expected, after 7 days of treatment with excess coconut oil, control group showed significant increase in total cholesterol, TG, LDL, VLDL and HDL cholesterol; whereas, the reference group showed around 7% decrease in total cholesterol, 66% increase in TG, 13% decrease in LDL, 9% decrease in VLDL and 94% increase in HDL cholesterol.

**Figure 10:**
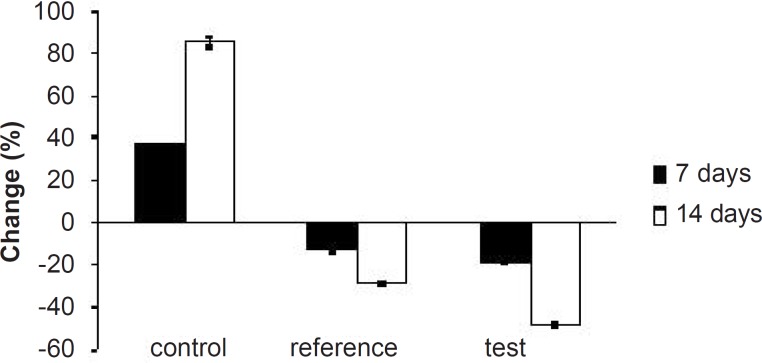
Percentage of changes in LDL levels of experimental groups at different time intervals

Interestingly, test group in comparison with the reference one presented 2.5-fold decrease in total cholesterol, 1.5-fold increase in TG, 1.4-fold decrease in LDL, 2.4-fold decrease in VLDL and 1.3-fold increase in HDL-cholesterol. After 14 days of similar treatment, control group displayed further increase in all the lipid levels; whereas, reference group showed further decrease in total cholesterol, LDL and VLDL, significant increase in TG and slight increase in HDL cholesterol. Test group on the other hand, presented 1.5-fold decrease in total cholesterol, negligible increase in TG, 1.4-fold decrease in LDL, 1.6-fold decrease in VLDL and 1.3-fold increase in HDL-cholesterol compared to the reference group. Thus, at the end of 14 days of the study, FD_2_ was better in reducing the total cholesterol, TG and LDL levels than the pure SIM (Figure 8-10). This could be primarily attributed to the improved solubility and dissolution associated with the amorphization of the drug (10). Moreover, pharmacodynamic evaluation in rats also justified the improvement in therapeutic efficacy of optimized FDs over the pure SIM. This may attributed to the improved solubility and dissolution associated with the amorphization of the drug. Since the FD_2_ batch elicited superior results, it can be proposed as a good candidate for systemic product development.

## Conclusions

The fused dispersions were prepared and a low level of X_1_ and a high level of X_2_ were optimized for obtaining a higher dissolution of SIM from SIM FDs. On increasing the melt to cool drug temperature, t_90%_ was increased which led to the improvement of FD_2_ batch dissolution rate with maximum drug release (99.63%) in 120 min. Moreover physicochemical characterizations and pharmacodynamic evaluation in rats justified the improvement in therapeutic efficacy of optimized FDs over the pure SIM. Since the FD_2_ batch elicited the superior results, it can be proposed as a good candidate for the systemic product development.
